# Comprehensive Mutational Landscape of Yeast Mutator Strains Reveals the Genetic Basis of Mutational Signatures in Cancer

**DOI:** 10.1093/molbev/msaf252

**Published:** 2025-10-06

**Authors:** Lei Liu, Danyang Sun, Haoxuan Liu

**Affiliations:** Center for Evolutionary & Organismal Biology and the Fourth Affiliated Hospital of Zhejiang University, Zhejiang University School of Medicine, 866 Yuhangtang Road, Hangzhou 310058, People's Republic of China; Center for Evolutionary & Organismal Biology and the Fourth Affiliated Hospital of Zhejiang University, Zhejiang University School of Medicine, 866 Yuhangtang Road, Hangzhou 310058, People's Republic of China; Center for Evolutionary & Organismal Biology and the Fourth Affiliated Hospital of Zhejiang University, Zhejiang University School of Medicine, 866 Yuhangtang Road, Hangzhou 310058, People's Republic of China

**Keywords:** mutation rate, mutation spectrum, mutation signature, yeast, gene deletion

## Abstract

Spontaneous mutation rates and spectra are influenced by an intricate interplay of processes including DNA replication, proofreading, and diverse DNA damage repair pathways. Although significant progress has been made in characterizing the functions of individual genes involved in these processes, their direct effects on mutation rates and spectra remain unclear. In this study, we employed a systematic gene knockout approach coupled with mutation accumulation (MA) experiments and whole-genome sequencing (WGS) to investigate the mutational landscape of *Saccharomyces cerevisiae*. We targeted 136 gene-deletion strains encompassing nearly all known genes associated with DNA replication and repair. Analysis of 1,081 MA lines revealed that 114 of the 136 genes significantly influenced at least one type of mutation rate. Furthermore, deletions of specific genes led to marked shifts in mutational biases and spectra, with some deletions amplifying existing biases and others reversing them entirely. In contrast, mitochondrial mutation rates were notably less affected, with no significant impact detected. Importantly, comparative analysis revealed striking similarities between yeast mutational spectrum and those observed in human cancers with the same pathway deficiencies, suggesting conserved functional roles across species. In conclusion, our findings provided critical insights into the genetic underpinnings of these signatures and underscoring the utility of yeast as a model for studying the molecular basis of cancer-associated mutational processes.

## Introduction

Spontaneous mutations are the fundamental source of all genetic variation, fueling evolution and biodiversity (Baer et al. 2007; Scally and Durbin 2012). The rate at which these mutations occur, commonly measured as mutations per base per generation, is a key parameter in molecular evolution (Gillespie 1984). Research has shown that mutation rates not only influence evolutionary processes but also respond to natural selection and evolve as a trait (Lynch et al. 2016; Liu and Zhang 2021). At the molecular level, mutation rates are governed by several factors, including the accuracy of DNA replication, the effectiveness of proofreading mechanisms, and the efficiency of various DNA damage repair pathways (Loeb and Monnat 2008; Boiteux and Jinks-Robertson 2013). Given their importance, the genes regulating these processes have been extensively studied. In the model organism *Saccharomyces cerevisiae*, 158 genes have been identified as playing crucial roles in DNA replication and repair (Huang et al. 2003; Boiteux and Jinks-Robertson 2013; Serero et al. 2014; Stirling et al. 2014; Jiang et al. 2021). Notably, the functions of these genes and the underlying mechanisms are highly conserved between yeast and humans (Boiteux and Jinks-Robertson 2013), underscoring the relevance of yeast-based research for understanding human genetic processes.

An important characteristic of spontaneous mutations is their spectrum, which refers to the relative frequencies of different mutation types (Horton and Taylor 2023). For example, the six possible types of single nucleotide substitutions (SBS) occur at uneven rates: transitions are generally more frequent than transversions, and GC-to-AT mutations are more common than AT-to-GC mutations. The latter bias is thought to be a major determinant of genomic GC content (Hershberg and Petrov 2010). The SBS spectrum holds particular significance in cancer research, where it has been studied in greater detail. By accounting for the adjacent 5′ and 3′ nucleotide context of each SBS, the six basic types are expanded into 96 distinct mutation categories. Specific combinations of the 96 categories are known as SBS signatures (Alexandrov et al. 2020). These signatures serve as molecular fingerprints of the mutational processes that have contributed to cancer development, including environmental exposures and genetic defects (Alexandrov et al. 2013; Ng et al. 2017; Liu et al. 2021; Boot et al. 2022; Chen et al. 2024). As such, they have considerable potential for improving cancer diagnosis and guiding treatment strategies. For instance, SBS6 and SBS15 are strongly associated with mismatch repair (MMR) deficiencies. When these signatures are detected in a patient, it likely indicates loss-of-function mutations in the MMR pathway, suggesting that the patient could benefit from treatments like immune checkpoint inhibitors, such as PD-1 inhibitors (Le et al. 2015, 2017). Similarly, SBS3 is linked to homologous recombination (HR) repair deficiencies, and patients with this defect often respond well to PARP inhibitors (Keung et al. 2019; Chopra et al. 2020). Despite the critical importance of SBS signatures, the genetic and environmental origins of most of the 86 identified signatures remain unclear. This highlights the need for further research to uncover the mechanisms underlying these mutational patterns and their implications for human health.

A central question in studying the function of genes involved in DNA replication and repair is how these genes influence spontaneous mutation rates and spectra.(Huang et al. 2003) One common method to address this is to delete a gene and observe how its absence alters mutation rates and spectra (Huang et al. 2003; Serero et al. 2014). Traditionally, mutation rates in yeast have been measured using reporter assays, which track the rate of loss-of-function mutations in a single gene. Using this method, Huang et al. identified 33 mutator strains from 4,847 yeast strains in the yeast gene-knockout collection (YKOC) (Huang et al. 2003). While efficient for large-scale screening, this method provides limited information about genome-wide mutation rates and spectra. To overcome this limitation, Puddu et al. leveraged the YKOC to examine repetitive DNA (rDNA) elements by sequencing the genomes of 4,732 strains, identifying genes involved in maintaining rDNA copy number (Puddu et al. 2019). Although these studies are effective for identifying genes that influence specific mutation types, they leave unanswered questions about the genes that affect more common mutation types, such as single nucleotide variations (SNVs) and small insertions/deletions (indels). These mutation types are difficult to study due to their low occurrence rates and typically require labor-intensive methods such as mutation accumulation (MA) experiments.

In an MA experiment, a randomly selected colony is streaked onto a new plate repeatedly over approximately 1,000 cell divisions. By continuously propagating single-cell-derived colonies, the effective population size is kept extremely small, minimizing the effects of natural selection and allowing spontaneous mutations to accumulate (Halligan and Keightley 2009). Although this approach is time-consuming, it enables the detection of rare SNVs and indels, preserving a comprehensive mutational record. Despite its challenges, MA combined with whole-genome sequencing (WGS) has been successfully employed to explore the mutational landscapes of yeast mutator strains. For example, one study established 44 MA lines from nine single- or double-gene-deletion strains and found significant changes in mutation rates and spectra across most strains after sequencing (Serero et al. 2014). Another study applied MA and WGS to 68 lines, including the wild type, two single-gene-deletion strains, and nine strains with mutator alleles (Stirling et al. 2014). This study similarly revealed substantial variation in mutation rates and spectra among mutant strains.

Together, these studies demonstrate the potential of MA and WGS to uncover genome-wide mutational effects of gene deletions. Despite these advances, prior studies have examined only a small fraction of the genes involved in DNA replication and repair. To address this gap, we conducted a large-scale study involving 1,081 MA lines of 136 yeast gene-deletion strains, covering nearly all known replication and repair genes. Our results revealed extensive variations in mutation rates and spectra across these strains, with pathway-specific effects in several DNA repair pathways. Strikingly, we found significant similarities in SBS signatures between yeast and human cancers with the same pathway deficiencies, highlighting conserved mechanisms and providing insights into the genetic basis of cancer-associated mutational signatures.

## Results

### Establishment and Sequencing of MA Lines

**Fig. 1.**
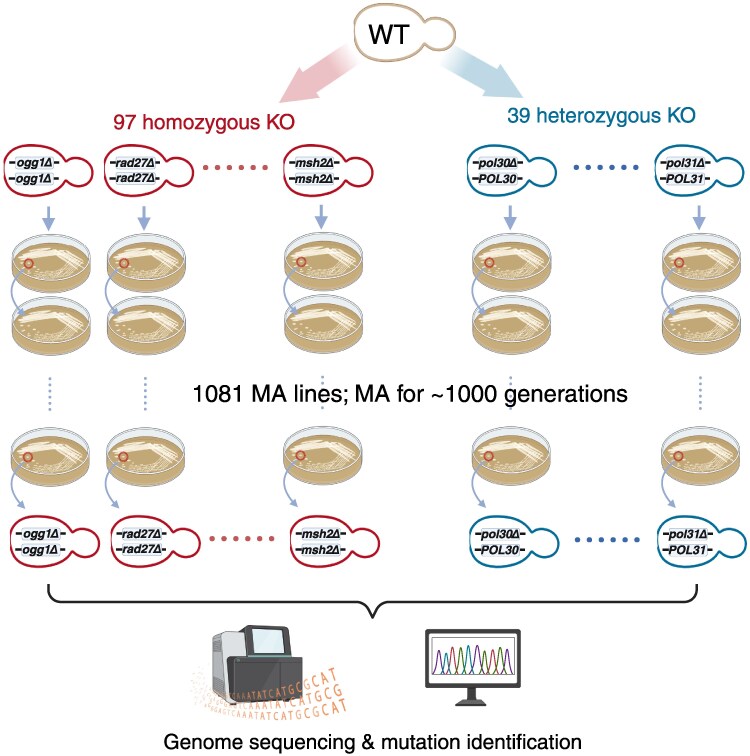
Schematic illustration of the study design. This study involved 136 KO strains derived from the wildtype BY4743. Among these, 97 strains were homozygous KOs (indicated in red on the left), and 39 strains were heterozygous KOs (indicated in blue on the right). The MA experiment spanned approximately 1,000 generations. In total, 1,081 MA lines underwent whole-genome sequencing and subsequent analysis.

To investigate the genetic basis of mutation rates and spectra, we focused on 159 genes associated with DNA replication and repair. The majority of these genes are non-essential, so we first screened the diploid set of the YKOC, obtaining 97 strains with homozygous deletions. The remaining genes are essential and could not be completely knocked out. For these, we created heterozygous knockouts (KOs) and successfully generated 39 heterozygous KO strains (Fig. 1 and Table S1). A subset of genes could not tolerate even heterozygous deletions, likely due to haploinsufficiency (Deutschbauer et al. 2005), and were excluded from further analysis. In total, we obtained 136 yeast KO strains, representing genes from all six major DNA repair pathways. This included 11 genes from the base excision repair (BER) pathway, 20 from the nucleotide excision repair pathway, 11 from the mismatch repair (MMR) pathway, 15 involved in post-replication repair (PRR), one gene associated with non-homologous end joining, and 71 additional genes involved in DNA replication and repair. Of these, 130 genes have functionally conserved counterparts in the human genome, emphasizing their broader biological relevance (Table S2).

To create a detailed mutational landscape for each KO strain, we established eight MA lines per strain, allowing mutations to accumulate over approximately 1,000 generations. Occasionally, some MA lines were lost due to contamination or lethal mutations. Finally, 1,081 MA lines were available for WGS. Both the ancestral strains and their corresponding MA lines were sequenced at high depth (average coverage >80×). To confirm the KOs, we analyzed sequencing read depth. Homozygous KOs were expected to show zero coverage over the deleted region, while heterozygous KOs were expected to show approximately 50% of the genome-wide average depth. These patterns were consistently observed across all KO strains, verifying the accuracy of the gene deletions (Fig. S1).

### Widespread Effects of Gene Deletions on Mutation Rates

Through WGS, we identified four types of mutations: SNVs, small insertions/deletions (indels ≤50 nucleotides), segmental duplications/deletions (>10 kb), and whole chromosomal gains/losses. SNVs and indels were detected through standard read mapping and variant calling, while large-scale mutations were identified via changes in read depth (Fig. S2; see Methods for details).

**Fig. 2.**
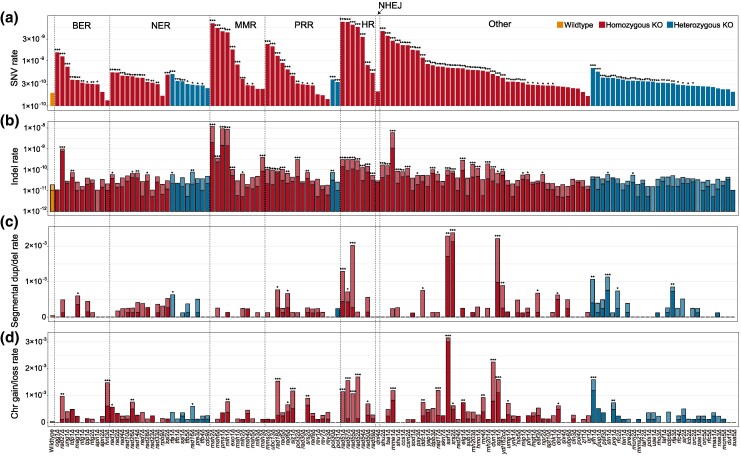
Variation in the four types of mutation rates across the 136 strains. a) Single-nucleotide variant (SNV) rates are shown for the wild-type strain and the 136 KO strains, with the *y*-axis displayed in log_10_ scale. b) Insertion and deletion (indel) rates are presented for the wild-type and 136 KO strains. Darker shades indicate insertion rates, while lighter shades represent deletion rates. The *y*-axis is in log_10_ scale. c) Rates of segmental duplications and deletions are shown with the *y*-axis in linear scale. Darker shades correspond to segmental duplication rates, while lighter shades represent segmental deletion rates. (d) Rates of chromosomal gains and losses are shown with the *y*-axis in linear scale. Darker shades indicating chromosomal gains and lighter shades representing chromosomal losses. In all panels, the first bar (orange) represents the wild-type strain, red bars denote homozygous KO strains, and blue bars indicate heterozygous KO strains. Strain names are displayed along the *x*-axis, organized by pathway, KO type, and SNV rate. Statistical significance between each KO strain and the wild-type strain was assessed using the Poisson test. Significance levels after Benjamini–Hochberg multiple test correction are denoted as follows: *P* < 0.05 (*), *P* < 0.01 (**), and *P* < 0.001 (***).

In total, we identified 31,329 mutations, with SNVs being the most frequent (22,535), followed by indels (8,116). Large-scale mutations were less common, with 275 segmental duplications/deletions and 403 chromosomal gains/losses (See Data S1 for a list of all mutations). Using the wild-type (WT) strain BY4743 as a control, we systematically evaluated how each gene KO affected mutation rates. Of the 136 KO strains, 111 exhibited significantly higher SNV rates than WT (Poisson test, *P* < 0.05 after Benjamini-Hochberg multiple test correction; Fig. 2a). The most dramatic increases were observed in *rad51Δ* and *rad57Δ* strains, where SNV rates were approximately 37-fold higher than WT, exceeding 7 × 10^−9^ per site per generation. Interestingly, two strains (*hnt3Δ* and *rev3Δ*) showed reduced SNV rates, at 68%–73% of WT levels. Although these reductions were not statistically significant using the Poisson test, they reached significance when analyzed with the Wilcoxon rank-sum test (*P* < 0.05). While the reason for *hnt3Δ*'s lower SNV rate is unclear, *REV3* is known to cause an antimutator phenotype when mutated (Roche et al. 1994). *REV3* encodes the catalytic subunit of error-prone DNA polymerase ζ (Friedberg et al. 2001), which may shift cells toward higher-fidelity repair pathways in its absence. Indel rates were significantly elevated in 52 KO strains (Poisson test, *P* < 0.05 after Benjamini-Hochberg multiple test correction; Fig. 2b), with *msh2Δ*, *msh6Δ*, and *mlh1Δ* displaying the highest rates—463- to 628-fold greater than WT. These three genes, all part of the mismatch repair (MMR) pathway, highlight its critical role in suppressing indel mutations. The magnitude of the increase in indel rates was far greater than the increase in SNV rates, further emphasizing the importance of MMR in maintaining genome stability.

For large-scale mutations, 18 KO strains showed significantly elevated rates of segmental duplications/deletions, and 28 KO strains had increased chromosomal gain/loss rates (Poisson test, *P* < 0.05 after Benjamini-Hochberg multiple test correction; Fig. 2c and 2d). The largest increases in segmental duplication/deletion rates were observed in *xrs2Δ*, *asf1Δ*, and *sgs1Δ* strains, with rates 53–57 times higher than WT. These genes are primarily involved in double-strand break repair, chromosomal recombination or Ty1 transposition regulation (Bressan et al. 1999; Rockmill et al. 2003; Tsabar et al. 2016). Given the established role of LTR retrotransposons in driving genomic structural variations (Bourque et al. 2018), we investigated the association between the breakpoints of segmental duplications/deletions and the annotated positions of LTR retrotransposons in the yeast genome. We found compelling evidence supporting transposon-mediated segmental duplications/deletions. Specifically, 63% of breakpoints were located within a close vicinity (<2 kb) of an LTR retrotransposon—a proportion significantly higher than random expectation (2.95% of 10,000 randomly selected genomic positions fall within 2 kb of an LTR retrotransposon; *χ*² test, *P* < 0.001). Remarkably, *ASF1*, a gene also involved in chromatin assembly and Ty1 transposition regulation (Nyswaner et al. 2008), exhibited the highest chromosomal gain/loss rate when deleted. These findings highlight the critical roles of these genes in maintaining genomic stability and their involvement in transposon-mediated structural variations.

Notably, 30 of the 39 heterozygous KO strains exhibited at least one type of elevated mutation rate, a proportion comparable to that observed in homozygous KO strains. This suggests that reduced expression levels of these genes alone can exert a mutagenic effect. Similarly, previous studies have demonstrated that reduced expression of *POL1*, *POL2*, or *POL3* in budding yeast induces genomic instability, leading to increased rates of mitotic recombination, aneuploidy, and de novo mutations (Song et al. 2014; Zheng et al. 2016; Zhang et al. 2022). These findings highlight the critical role of gene dosage in maintaining genomic stability.

The four mutation types were not independent but correlated. Across the 136 KO strains, the strongest correlations were observed between SNVs and indels (Pearson's *r* = 0.49, *P* = 9.8 × 10^−10^) and between segmental duplications/deletions and chromosomal gains/losses (Pearson's *r* = 0.50, *P* = 8.4 × 10^−10^). However, correlations between small and large mutation rates were weaker (average Pearson's *r* = 0.097). Overall, 114 of the 136 KO strains significantly altered at least one type of mutation rate, highlighting the pervasive impact of these genes on genome stability.

Apart from genes in the canonical repair pathways, several genes unassigned to these pathways also significantly affect mutation rates upon deletion. Among these, the Shu complex genes, *SHU1*, *SHU2*, *CSM2*, and *PSY3*, stand out. Their deletion increases the SNV rate by an average of 14-fold. Previous studies have linked the Shu complex to the HR pathway (Martino and Bernstein 2016). If the Shu complex influences mutation rates solely through the HR pathway, its deletion should not further increase mutation rates when the HR pathway is already nonfunctional. To test this hypothesis, we first deleted *RAD57*, a key component of the HR pathway, and then deleted *SHU1* or *SHU2* in the Shu complex. We employed fluctuation tests to measure the effects of single and double deletions on mutation rates. Individual deletions of *RAD57*, *SHU1*, or *SHU2* each significantly increased mutation rates (all *P* < 0.001, likelihood-ratio tests; Fig. S3a). Importantly, deleting *SHU1* or *SHU2* in a *rad57Δ* background did not further increase mutation rates (all *P* > 0.05, likelihood-ratio tests; Fig. S3a). This indicates that the Shu complex regulates mutation rates through the HR pathway. Similarly, deletion of *DDC1*, which encodes a DNA damage checkpoint protein also associated with HR (Hong and Roeder 2002), produced the same single- and double-deletion pattern (Fig. S3a).

In addition, four genes involved in oxidative stress, *SOD1*, *CCS1*, *TSA1*, and *YAP1*, also showed strong effects on SNV rates. These genes, which form two physically interacting pairs (*SOD1–CCS1* (Wood and Thiele 2009) and *TSA1–YAP1* (Lee et al. 1999)), increased SNV rates by an average of 11-fold upon single deletion. This suggests that reactive oxygen species (ROS) are a major source of de novo mutations. To determine whether oxidative stress-induced damage is primarily repaired by a specific pathway, we deleted *SOD1* or *TSA1* in combination with key genes from four repair pathways: *OGG1* (BER), *MSH2* (MMR), *MMS2* (PRR), and *RAD57* (HR). Consistent with the results from MA combined with WGS, single deletions of any of these genes increased *CAN1* mutation rates (Fig. S3b). However, unlike the Shu complex, deletion of *SOD1* or *TSA1* further increased mutation rates in strains lacking *OGG1*, *MSH2*, *MMS2*, or *RAD57* (all *P* < 0.001, likelihood-ratio tests; Fig. S3b). This indicates that ROS-induced damage is not repaired by a single pathway but instead requires multiple repair mechanisms.

### Gene Deletions Have Minimal Impact on Mitochondrial Mutation Rates

The dataset we generated also allowed us to investigate mutation rates in the mitochondrial (MT) genome. By calculating the relative MT read depth compared to the nuclear genome, we first confirmed the presence of MT DNA in most KO strains before MA, with an average copy number of 13.8 (Fig. S4a). After MA, MT copy numbers declined significantly to an average of 3.97 (Paired *t*-test, *P* = 1.7 × 10^−47^; Fig. S4b), indicating reduced selective pressures on MT maintenance during this process.

Due to the simpler damage repair mechanisms and smaller number of repair-associated genes in the MT genome (Kazak et al. 2012), many gene deletions were not expected to impact MT mutation rates. Indeed, no significant differences were found between any KO strains and the WT (Poisson test, *P* > 0.05 after Benjamini-Hochberg multiple test correction; Fig. S4c and S4d). These findings confirm that the maintenance of the MT genome involves fewer repair genes compared to the nuclear genome.

### Mutation Biases Can be Reversed and Spectra Significantly Altered by Gene Deletions

**Fig. 3.**
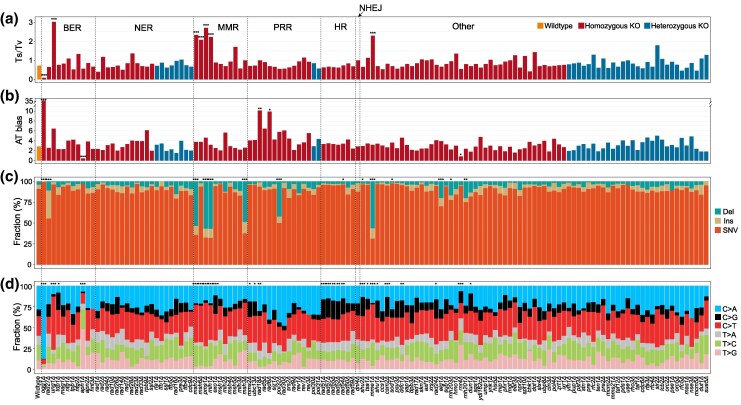
Variations in mutational biases and spectra across the KO strains. a) Levels of transition bias (Ts/Tv) across the wildtype and 136 KO strains. Transition bias is calculated as the number of transition mutations divided by the number of transversion mutations. b) Levels of AT bias across the wildtype and 136 KO strains. AT bias is defined as the rate of GC-to-AT mutations divided by the rate of AT-to-GC mutations. c) Proportions of deletions, insertions, and single-nucleotide variants (SNVs) across the wildtype and 136 KO strains. d) Relative frequencies of the six types of SNVs across the wildtype and 136 KO strains. Strains are arranged in the same order as in Fig. 2, organized by pathway, KO type, and SNV rate. Statistical comparisons of ratios and fractions between each KO strain and the wild-type strain were performed using the *χ*² test. Significance levels are indicated as follows: *P* < 0.05 (*), *P* < 0.01 (**), and *P* < 0.001 (***).

SNV mutations exhibit two biases that are nearly universal across species: transition bias (Lee et al. 2012) and AT bias (Hershberg and Petrov 2010). Transition bias refers to the tendency for the transition/transversion (Ts/Tv) ratio to be higher than the random expectation of 0.5. In our study, the Ts/Tv ratio was significantly increased in six KO strains compared to the WT (*χ*² test, *P* < 0.001 after Benjamini-Hochberg multiple test correction; Fig. 3a). The highest ratio was observed in the *ung1Δ* strain, where the proportion of C > T mutations increased from 23% in the WT to 64%. *UNG1*, a uracil-DNA glycosylase, repairs uracil residues in DNA formed by spontaneous cytosine deamination (Impellizzeri et al. 1991), explaining this pronounced bias. Interestingly, reversed transition bias was observed in the *ogg1Δ* strain, where the Ts/Tv ratio dropped to 0.08, far below the 0.5 threshold (Binomial test, *P* = 1.8 × 10^−47^). *OGG1*, a glycosylase, excises 7,8-dihydro-8-oxoguanine residues opposite C or T in DNA (van der Kemp et al. 1996). When *OGG1* is deleted, the proportion of C > A mutations rose dramatically from 29% in WT to 88%. This elevated frequency of C > A mutations also led to an increase in AT bias in the *ogg1Δ* strain, with the μ_GC→AT_/*μ*_AT→GC_ ratio exceeding 35, much higher than in WT (Fig. 3b). This finding is consistent with the report of elevated C > A mutation rates in natural yeast isolates harboring mutator allele of *OGG1* (Jiang et al. 2021). Beyond *ogg1Δ*, AT bias was significantly elevated in two additional KO strains (*rad18Δ*and *rad6Δ*) and significantly reduced in two strains (*apn1Δ* and *rnr4Δ*). Notably, AT bias reversed entirely to GC bias in the *apn1Δ* strain, where the μ_GC→AT_/*μ*_AT→GC_ ratio was 0.46, significantly lower than 1 (Binomial test, *P* = 0.014).

In the WT and most KO strains, SNVs were the most common mutation type. However, indel mutation rates were significantly increased and surpassed SNV rates in five KO strains (Fig. 3c). Four of these strains involved deletions of genes from the MMR pathway, further emphasizing the pathway's essential role in repairing indels. Furthermore, SNVs are categorized into six types based on their nucleotide changes, and the relative frequencies of these types define the SNV spectrum. Gene deletions had a significant impact on SNV spectra in 26 of the 136 KO strains (*χ*² test, *P* < 0.05 after Benjamini-Hochberg multiple test correction; Fig. 3d). These 26 genes spanned multiple DNA repair pathways, suggesting that different pathways exhibit specific preferences for repairing certain types of SNVs.

### Genes From the HR Pathway Exhibit Strikingly Similar Effect on Mutational Spectra Upon Deletion

**Fig. 4.**
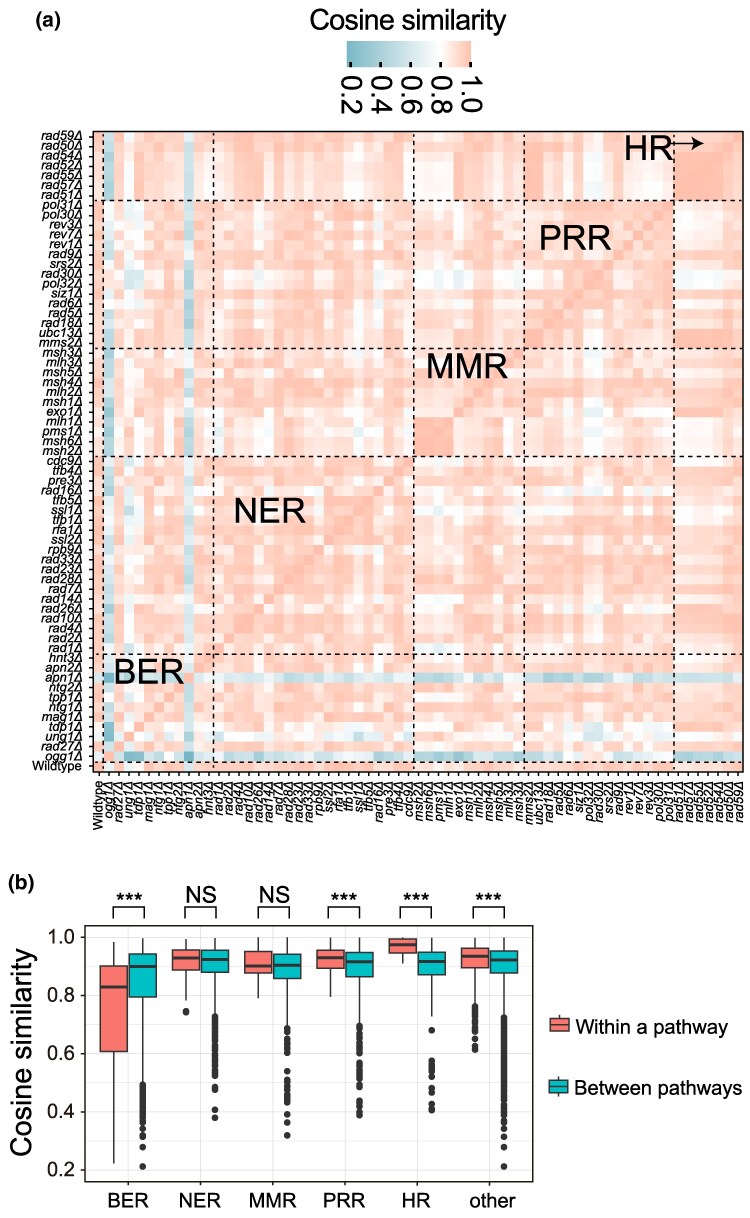
Within- and between-pathway comparisons of correlation in SBS signatures among KO strains. a) Cosine similarity of the relative frequencies of the six types of SNVs were computed among each KO strains and the WT. Pink indicating higher levels of similarity while blue indicating lower levels of similarity. Pathways are delineated by vertical and horizontal dashed lines. b) Comparison of cosine similarity within and between pathways. Cosine similarities were compared between strains with deletions in the same pathway and those with deletions in different pathways. Data are presented as box plots for each group. The lower and upper edges of a box represent the first (qu1) and third quartiles (qu3), respectively, and the horizontal line inside the box indicates the median (md). The whiskers extend to the most extreme values inside inner fences, md ± 1.5(qu3–qu1), and the dots represent values outside the inner fences (outliers). Statistical significance between the two groups in each comparison was assessed using the Wilcoxon rank-sum test. Significance levels are denoted as follows: *P* < 0.001 (***).

The functional specificity of DNA repair pathways often results in distinct mutational spectra when these pathways are deficient. However, as shown earlier, genes within the same pathway may vary in their functional importance, leading to differing impacts on mutation rates and spectra when deleted. To systematically access the pathway-specific impact on mutational spectra, we computed the cosine similarity of the relative frequencies of the six types of SNVs among all 137 strains, including the WT and 136 KO strains (Fig. 4a and Fig. S5). Genes from the BER pathway exhibit significantly lower within-pathway cosine similarity (Wilcoxon rank-sum test, *P* < 0.001; Fig. 4b), indicating that their deletions lead to diverse functional consequences. Notably, *ogg1Δ* and *apn1Δ* showed the lowest between-strain cosine similarity (0.56 and 0.60, respectively), with *ogg1Δ* primarily driving an increase in C > A mutations and *apn1Δ* leading to elevated T > G mutations. In contrast, within-pathway cosine similarity is generally higher for other repair pathways, with the HR pathway exhibiting the most striking effect. The average within-pathway cosine similarity for HR gene deletions reaches 0.97, significantly higher than that observed between pathways (Wilcoxon rank-sum test, *P* < 0.001; Fig. 4b), highlighting the remarkably consistent mutational spectra resulting from HR gene deletions. Collectively, these findings suggest that gene deletions within the same pathway do not always produce similar mutational spectra. While BER gene deletions often lead to distinct mutational profiles, HR gene deletions tend to produce highly consistent mutational spectra.

### Conserved Effects of Pathway Deficiency on SBS Signatures Between Yeast and Humans

To examine the effects of gene deletion on mutational spectra across species, we conducted a systematic comparison with previous studies on budding yeast (Serero et al. 2014; Stirling et al. 2014; Loeillet et al. 2020), *C. elegans* (Meier et al. 2018), and human cell line (Zou et al. 2021). Specifically, we computed the cosine similarity of the 96 SBS types between our study and these previous studies. We found significantly higher cosine similarity between yeast strains with the same gene deletions. The average cosine similarity for the nine comparisons involving the same gene deletions was 0.84, substantially higher than that between different genes (averaging 0.55; Wilcoxon rank-sum test, *P* < 0.001; Fig. S6a). Furthermore, near-identical SBS spectra were observed for gene-deletion strains with a high accumulation of mutations, such as *msh2Δ* and *rad51Δ*, with their cosine similarity averaging 0.96. This finding underscores the reproducibility and robustness of the data generated in the current study.

Additionally, higher cosine similarity was also observed between our study and two gene deletions in the MMR pathway in *C. elegans*. The cosine similarity between the orthologous gene deletions in yeast and *C. elegans* averaged 0.76, significantly higher than the average similarity between different genes (0.42; Fig. S6b). Despite humans diverging from budding yeast over a billion years ago (Douzery et al. 2004), high cosine similarity was still observed between deletions of two key genes in the BER pathway. Specifically, the cosine similarity for the *ogg1Δ* deletion between human and yeast strains was 0.89, and 0.79 for the *ung1Δ* deletion, both of which are substantially higher than the between-gene average of 0.41. Similarly, high cosine similarities were observed for three gene deletions within the MMR pathway, with the average between-species cosine similarity reaching 0.92 for the *msh2Δ*, *msh6Δ*, and *mlh1Δ* deletions (Fig. S6c).

The conserved effects of gene deletions across species prompted us to utilize yeast as a model organism to explore SBS signatures in cancer. SBS signatures are extensively studied in cancer due to their importance in diagnosis and treatment (Levatić et al. 2022; Thatikonda et al. 2023; Jayakrishnan et al. 2024). These signatures often arise from two primary sources: genetic defects in DNA replication and repair genes, and environmental factors (Alexandrov et al. 2013). Despite the identification of 86 SBS signatures, the etiology of more than half (44 signatures) remains unknown. A major challenge in identifying genetic causes is the sheer number of mutations in cancer genomes, which makes it difficult to pinpoint causal mutations. The dataset we generated, with only one gene knocked out per yeast strain, offers a unique opportunity to disentangle the genetic basis of SBS signatures.

**Fig. 5.**
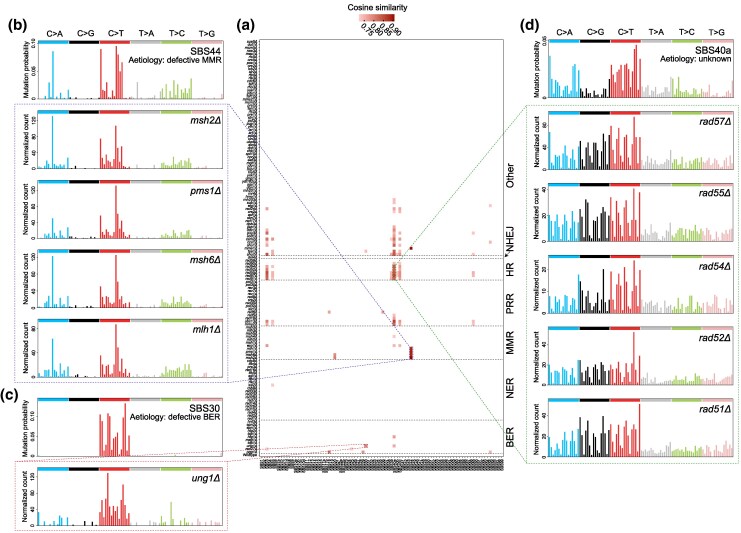
Conserved effects of repair pathway deficiencies between yeast and humans. a) Heatmap of cosine similarities in SBS signatures between yeast and human cancer. The heatmap illustrates top 100 highest cosine similarities between 86 established SBS signatures in human cancer (*x*-axis) and 137 yeast strains (*y*-axis), including the wild-type and 136 KO strains. Darker colors indicate higher similarities. b) Comparison of SBS Signature SBS44 (human) and MMR-Deficient Yeast Strains. The bar plots display the relative frequencies of the 96 types of SNVs (considering adjacent 5′ and 3′ nucleotides) in SBS44, a human SBS signature associated with defective mismatch repair (MMR), and four yeast strains with MMR pathway deficiencies. c) Comparison of SBS Signature SBS30 (human) and a BER-deficient yeast strain. The bar plots compare the relative frequencies of SNVs in SBS30, a human SBS signature linked to defective base excision repair (BER), and a yeast strain with BER pathway deficiencies. d) Comparison of SBS Signature SBS40a (human) and HR-deficient yeast strains. The bar plots compare SBS40a, a human SBS signature with an unknown etiology, to the SNV profiles of five yeast strains with deficiencies in the HR pathway.

To explore whether the same pathway deficiencies produce similar SBS signatures in yeast and humans, we computed cosine similarities between SBS mutational spectrum from each yeast KO strain and annotated cancer signatures. Of the top 100 highest cosine similarities, the WT strain showed no correlations with any cancer signatures, suggesting that spontaneous mutations in a WT genetic background lack preference for cancer-associated patterns. In contrast, high similarities were frequently observed between KO strains and established cancer signatures, which the average cosine similarity reaching 0.78 for the top 100 associations (Fig. 5a and Table S3).

Cancer signatures are not mutually exclusive, and an SBS signature from a KO strain may correlate with multiple cancer signatures. Focusing on the 30 strongest associations, we found that 17 of these cancer signatures have known genetic etiologies. Remarkably, 10 of these associations involved KO strains with deletions in genes belonging to the same pathway as the human mutations, significantly more than expected by chance (on average, only 1.1 of the 17 associations involved KO strains with deletions in genes from the same pathway when pathways in yeast strains were shuffled; *P* < 0.001 from 1,000 randomizations). This finding indicates that pathway deficiencies produce conserved effects on SBS signatures across yeast and humans. For example, SBS44, a signature associated with defective MMR, showed a strong correlation with four yeast strains deficient in MMR (Fig. 5b). Similarly, SBS30, which is linked to defects in BER, were closely associated with yeast strains lacking key BER components (Fig. 5c). Building on these clear parallels, we explored the genetic basis of cancer signatures with unknown etiologies. One such example is SBS40a, whose origin is currently unclear. However, our analysis revealed a strong association between SBS40a and yeast strains with HR deficiencies (Fig. 5d). These findings highlight the potential of yeast as a powerful model for studying cancer genetics. By leveraging the genetic simplicity and experimental control offered by yeast, we can gain valuable insights into the mechanisms underlying mutational signatures in human cancer, including those with previously unknown causes.

## Discussion

This study provides a detailed and comprehensive mutational landscape of yeast gene-deletion strains, offering new insights into the functional roles of DNA replication and repair pathways. By combining MA and WGS, we systematically examined the effects of 136 gene deletions on mutation rates, biases, and spectra. In doing so, we not only confirmed the essential roles of canonical pathways but also uncovered unexpected contributors and novel mechanistic relationships. Importantly, this dataset extends prior work (Meier et al. 2018; Zou et al. 2021) by demonstrating that conservation of mutational signatures is a general property across diverse pathways, while also highlighting cases of heterogeneity within pathways and newly revealed genetic interactions.

Our results demonstrate that 114 of the 136 gene deletions significantly altered at least one type of mutation rate, including SNVs, small indels, segmental duplications/deletions, and chromosomal gains/losses. The MMR pathway emerged as critical for suppressing indel rates, with deletions of *MSH2*, *MSH6*, and *MLH1* causing increases in indel rates of over 600-fold compared to the WT. Similarly, genes in the HR pathway, such as *RAD51* and *RAD57*, had the largest impact on SNV rates, showing 37-fold increases relative to WT. These findings highlight the essential roles of MMR and HR in maintaining genomic stability, consistent with previous reports (Serero et al. 2014), but extend these observations with comprehensive genome-wide data. Interestingly, some gene deletions led to unexpected decreases in mutation rates. For instance, *REV3*, encoding the catalytic subunit of the error-prone DNA polymerase ζ, exhibited reduced SNV rates, likely due to compensatory shifts toward higher-fidelity repair pathways. This finding underscores the complexity of DNA repair networks and suggests that certain gene deletions can trigger adaptive responses that mitigate mutational damage.

One of the most striking findings of this study is the conservation of mutational signatures between yeast and humans. For example, SBS44, a signature associated with defective MMR in human cancers, strongly correlated with yeast strains deficient in *MSH2*, *MSH6*, *MLH1*, and *PMS1*. Similarly, SBS30, linked to BER deficiencies in humans, were closely associated with yeast strains lacking *UNG1*. These results validate the functional conservation of DNA repair pathways across species and suggest that yeast can serve as a proxy for investigating the genetic basis of SBS signatures in human cancers. Moreover, our analysis shed light on previously uncharacterized cancer signatures. For example, SBS40a, whose etiology is currently unknown, was strongly associated with yeast strains deficient in HR repair, such as *RAD51* and *RAD52*. This finding suggests a potential link between HR defects and SBS40a in human cancers, suggesting that patients with exhibiting this signature could benefit from treatments such as PARP inhibitors. These inhibitors have demonstrated promising efficacy in cancers with HR deficiencies, as they exploit the reliance of HR-deficient cancer cells on PARP-mediated BER for survival (Keung et al. 2019). By selectively targeting cancer cells while sparing normal ones, PARP inhibitors offer a tailored therapeutic approach. This discovery establishes a foundation for further research into the genetic origins of SBS40a and its potential implications for personalized cancer treatment strategies.

Future studies in this field could expand by exploring gene-environment interactions and gene-gene interactions. In this study, the experimental conditions were based on a nutrient-rich, non-stressful medium (YDP). However, both mutation rates and spectra are known to be significantly influenced by environmental factors (Liu and Zhang 2019). Since human tumors often exist in more stressful and complex environments (Anderson and Simon 2020; de Visser and Joyce 2023), future research could address this limitation by incorporating stressors such as oxidative damage or by replicating the tumor microenvironment in yeast models. Additionally, while this study focused on single-gene deletions, many cancers involve intricate interactions between multiple genes. Extending this research to include combinatorial gene knockouts could offer a more comprehensive understanding of how interactions between DNA repair pathways shape mutational signatures. Such advancements would provide deeper insights into the genetic and environmental influences on mutational processes and their implications for cancer biology.

In summary, this study presents a comprehensive analysis of mutational patterns in yeast gene-deletion strains, providing valuable insights into the genetic basis of mutational signatures in cancer. The findings reveal that pathway-specific deficiencies generate distinct mutational profiles, which can serve as biomarkers for identifying DNA repair defects in cancer. This has significant clinical implications, as recognizing mutational signatures linked to MMR or HR deficiencies could inform personalized treatment strategies. For example, immune checkpoint inhibitors for MMR-deficient cancers or PARP inhibitors for HR-deficient tumors. Furthermore, the discovery of conserved mutational signatures between yeast and humans underscores the value of yeast as a cost-effective and high-throughput model for investigating mutational processes in cancer. By integrating yeast genetics with cancer genomics, this study establishes a robust foundation for future research focused on understanding and targeting DNA repair deficiencies in human diseases.

## Materials and Methods

### Strains and Medium

All strains used in this study were derived from the widely used laboratory strain BY4743. Of the 136 knockout (KO) strains analyzed, 97 were homozygous KO strains obtained from the YKOC, with each strain having a single gene disrupted. The homozygous diploid complete set of the YKOC was sourced from Invitrogen (catalog number 95401.H1R3). The remaining 39 strains were heterozygous KOs, with one allele of a target gene disrupted. Since the YKOC strains were generated by homologous recombination, replacing target genes with the KanMX4 cassette, a similar strategy was employed for constructing the heterozygous KO strains. For each gene, two 59-bp primers were designed. The inner 19 bp of each primer corresponded to the KanMX4 cassette (including its promoter), while the outer 40 bp was homologous to the flanking regions of the target gene's coding sequence (CDS). These primers were used in a PCR reaction with a plasmid containing the KanMX4 cassette as the template. The resulting PCR product served as a repair fragment during yeast transformation. Upon successful transformation, the target gene's CDS was replaced by the KanMX4 cassette and its promoter. Each repair fragment was introduced into yeast cells via high-efficiency transformation (Gietz and Schiestl 2007). Transformants were selected on YPD plates supplemented with 500 μg/mL kanamycin. Successful transformants were initially confirmed by PCR, using primers flanking the target gene to verify the expected product length. WGS was subsequently performed to validate the transformants. To quantify gene deletions, read depth at each genomic position was calculated using the DepthOfCoverage tool in the Genome Analysis Toolkit (GATK) (McKenna et al. 2010). The ratio of the average read depth of the deleted gene to the genomic average was determined. For homozygous KO strains, this ratio was <1%, while for heterozygous KO strains, it ranged from 40% to 60% (Fig. S1), confirming the expected deletions. Fluctuation test was carried out as previous described (Liu and Zhang 2021) and statistical analysis was performed using webSalvador (Zheng 2021).

### Mutation Accumulation

For each of the 136 KO strains, eight MA lines were established. All eight MA lines for a given strain were derived from a single ancestral colony, which was preserved and whole-genome sequenced to serve as a control for mutation identification. The MA experiment was conducted on solid YPD medium (1% yeast extract, 2% peptone, 2% dextrose, and 2% agar) at 30 °C. Every 48 h, a random colony from each MA line was selected and streaked onto a fresh plate. This process was repeated for 50 transfers, spanning a total of 100 days.

To estimate the number of cell divisions during the MA experiment, colony cell counts were performed after 48 h of growth for both the ancestral samples and the MA lines. The number of cell divisions required to form a colony was calculated as log_2_(number of cells in the colony), assuming exponential growth. The total number of generations was then estimated as:

Total generations = (Divisions per growth cycle for the ancestral sample + Divisions per growth cycle for the MA line) × 25

### Genome Sequencing and Mutation Identification

After the MA experiment, genomic DNA from the ancestral samples and MA lines was extracted using the S-16 Fully Automated Nucleic Acid Extractor from ABclonal (catalog number AI32201), which employs magnetic bead adsorption technology to enable the rapid extraction of high-quality nucleic acids from various biological samples. Library preparation and sequencing were performed by Repugene Technology (Hangzhou, China; https://www.repugene.com/). Genomic sequencing was conducted on the Illumina HiSeq platform, with a designated output of 1 Gb per sample.

Clean sequencing reads were mapped to the Saccharomyces cerevisiae reference genome (version R64-3-1) using bwa mem (Li and Durbin 2009) with default settings. The resulting SAM files were sorted using the SortSam program in Picard, and duplicate reads were marked with Picard's MarkDuplicates program (https://broadinstitute.github.io/picard/). Reads were filtered using Samtools (Li et al. 2009) with the following criteria: mapped reads with a mapping quality ≥20, and reads that were not unmapped, not mate-unmapped, not marked as duplicates, and not classified as secondary or supplementary alignments. The command used was:

“samtools view -b -h -q 20 -f 3 -F 4 -F 8 -F 256 -F 1024 -F 2048”.

Variants were identified using the HaplotypeCaller program in GATK, with the -A StrandBiasBySample option to include mapping directionality information. A mutation was called if it satisfied the following criteria:

1. The genotype at the site differed between the MA line and the ancestor, and the ancestral genotype was homozygous;
2. Both the ancestor and MA line had at least 10× coverage at the site;
3. The variant had a quality score of ≥50;
4. The mutant genotype was supported by at least three forward and three reverse reads;
5. For heterozygous variants, the allele frequency ranged between 30% and 70%;
6. The variant was located more than 10 kb from chromosomal ends.

Callable genomic sites for each sample were determined by counting regions with at least 10× coverage.

To identify large-scale mutations, read depth was calculated across non-overlapping 1-kb windows for each sample. Regions showing a change in read depth exceeding 30% of genomic average across 10 consecutive windows were classified as large-scale mutations. If the change extended across an entire chromosome, it was classified as a whole chromosomal gain or loss (Fig. S2).

To estimate the MT copy number, we determined the relative read depth of the MT genome compared to the nuclear genome and then multiplied the result by two, accounting for the diploid nature of the cells.

SNV and indel rates were calculated as total number of mutations from all replicates/number of replicates/average number of generations during MA/average number of callable sites, where callable sites were defined as number of genomic sites covered by at least 10 reads. Rates of segmental duplication/deletion and whole-chromosome gain/loss were calculated as total number of events from all replicates/number of replicates/average number of generations during MA.

### Analysis of SBS Signatures

Single Base Substitution (SBS) signatures (Alexandrov et al. 2020) (version 3.4) in human cancer is downloaded from https://cancer.sanger.ac.uk/signatures/sbs/. Signature visualization and associated analysis are performed using R package sigfit (Gori and Baez-Ortega 2020). To account for the differences in the distribution of genomic 3-mer sequences between yeast and humans, the yeast 3-mer mutation count is normalized to align with the 3-mer distribution observed in the human genome before computing the cosine similarity between the two species. This normalization is achieved by first dividing the yeast 3-mer mutation count by the relative proportion of the corresponding 3-mer in the yeast genome and then multiplying it by the proportion of the same 3-mer in the human genome. This adjustment ensures that the mutation counts reflect the 3-mer distribution of the human genome, enabling a more accurate comparison across species.

### Statistical Analysis and Figure Construction

Statistical analysis and figure construction were carried out in R. For the Poisson test comparing mutation rates between two strains, the ratio of the total number of observed mutations in the two strains is compared against the ratio of the expected opportunities for mutation, calculated as: number of replicates × average callable sites per replicate × number of generations during MA. The corresponding R code is:

poisson.test(

x = c(StrainA$SNV_count, StrainB$SNV_count),

T = c(StrainA$number_of_replicates * StrainA$number_of_generation * StrainA$average_callable_sites,

StrainB$number_of_replicates * StrainB$number_of_generation * StrainB$average_callable_sites)

)

For the Wilcoxon rank-sum test, the mutation rate of each replicate is treated as an independent data point. For example, if both strains A and B have eight replicates each, the eight mutation rate values from strain A are compared with the eight values from strain B using:

wilcox.test(mutation_rate$strainA, mutation_rate$strainB).

### Materials Availability

Yeast strains generated in this study are available upon request.

## Supplementary Material

msaf252_Supplementary_Data

## Data Availability

All sequencing reads generated in this study have been deposited in the China National Genomics Data Center under the project number PRJCA039741. Any additional information required to reanalyze the data reported in this paper is available from the lead contact upon request.
