# Supplementary material for: Comprehensive Mutational Landscape of Yeast Mutator Strains Reveals the Genetic Basis of Mutational Signatures in Cancer
Source: Mol Biol Evol. 2025 Oct 6;42(10):msaf252. doi: 10.1093/molbev/msaf252 (PMC12559997; doi:10.1093/molbev/msaf252)
Supplement: msaf252_Supplementary_Data [file msaf252_supplementary_data.zip › FigureS.revision2.pdf]

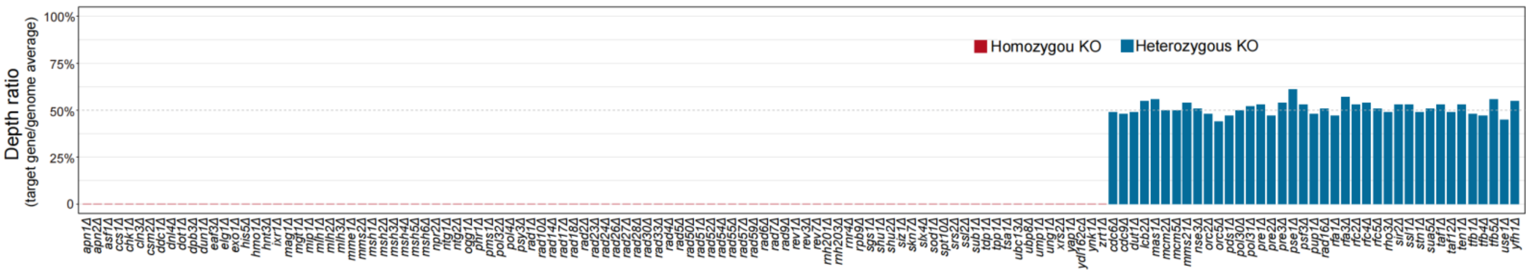

Figure S1. Ratio of read depth between deleted genes and genomic average. The figure displays the ratio of read depth for deleted genes relative to the genomic average across all KO strains. Homozygous KO strains are represented in red, while heterozygous KO strains are shown in blue.

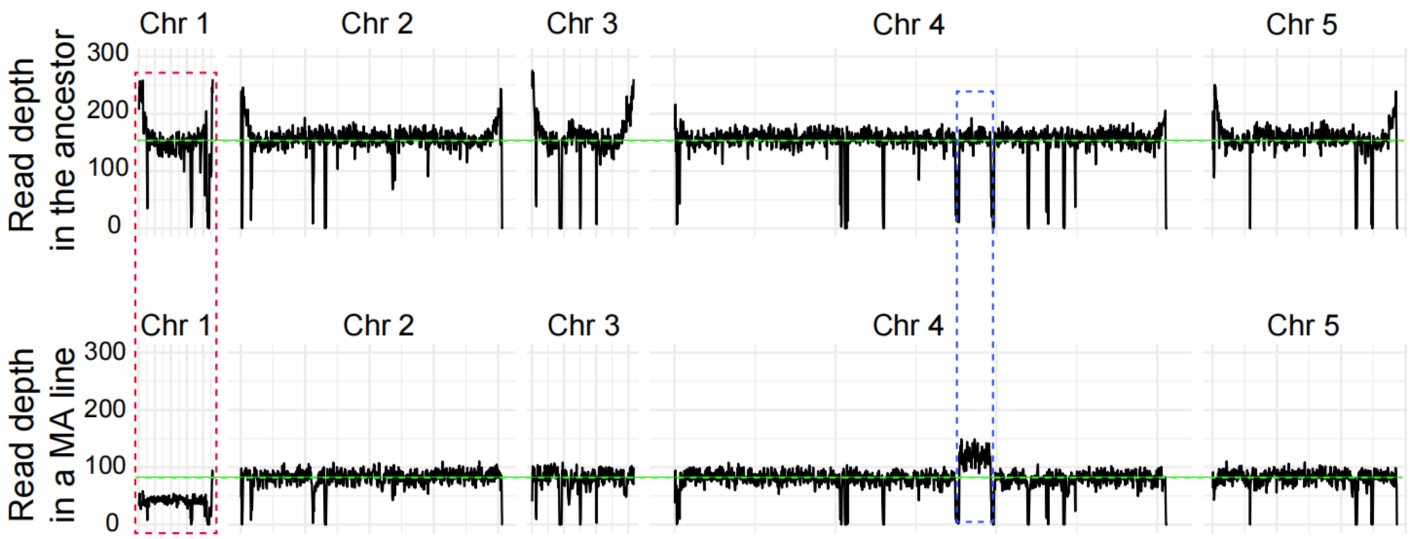

Figure S2. Illustration of segmental duplication/deletion and whole chromosomal gain/loss events. This figure depicts the read depth across the first five chromosomes in the ancestral *ump1Δ* strain (upper panel) and replicate 3 of the MA line (lower panel). Green dashed lines indicate the genomic average read depth. A whole chromosomal loss is highlighted by a red dashed box, while a segmental duplication event is marked by a blue dashed box.

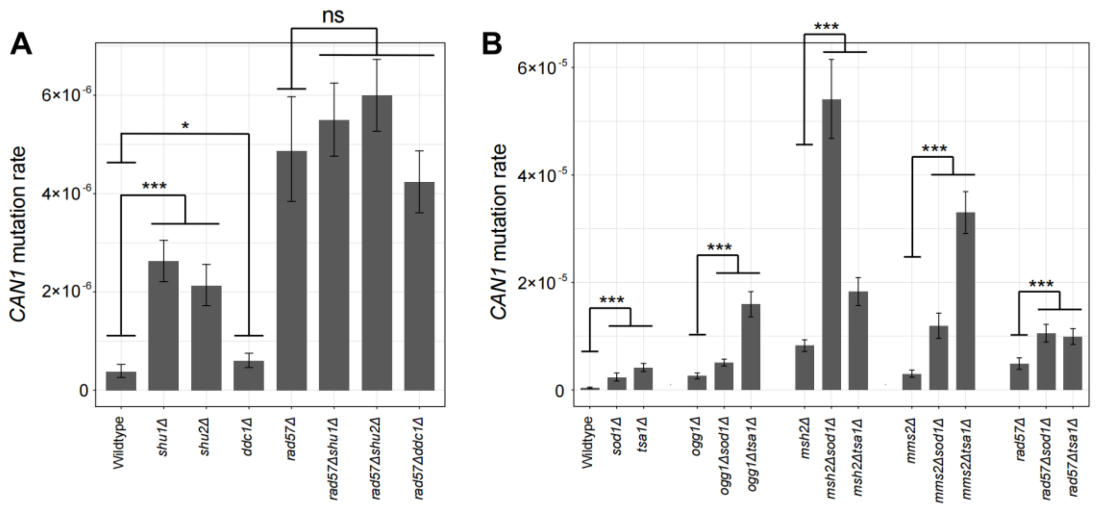

Figure S3. CAN1 mutation rates of single- and double-gene deletion strains. Statistical comparisons were performed using the likelihood ratio test implemented in webSalvador. Significance levels are indicated as follows: ns, not significant; $*P < 0.05$; $***P < 0.001$.

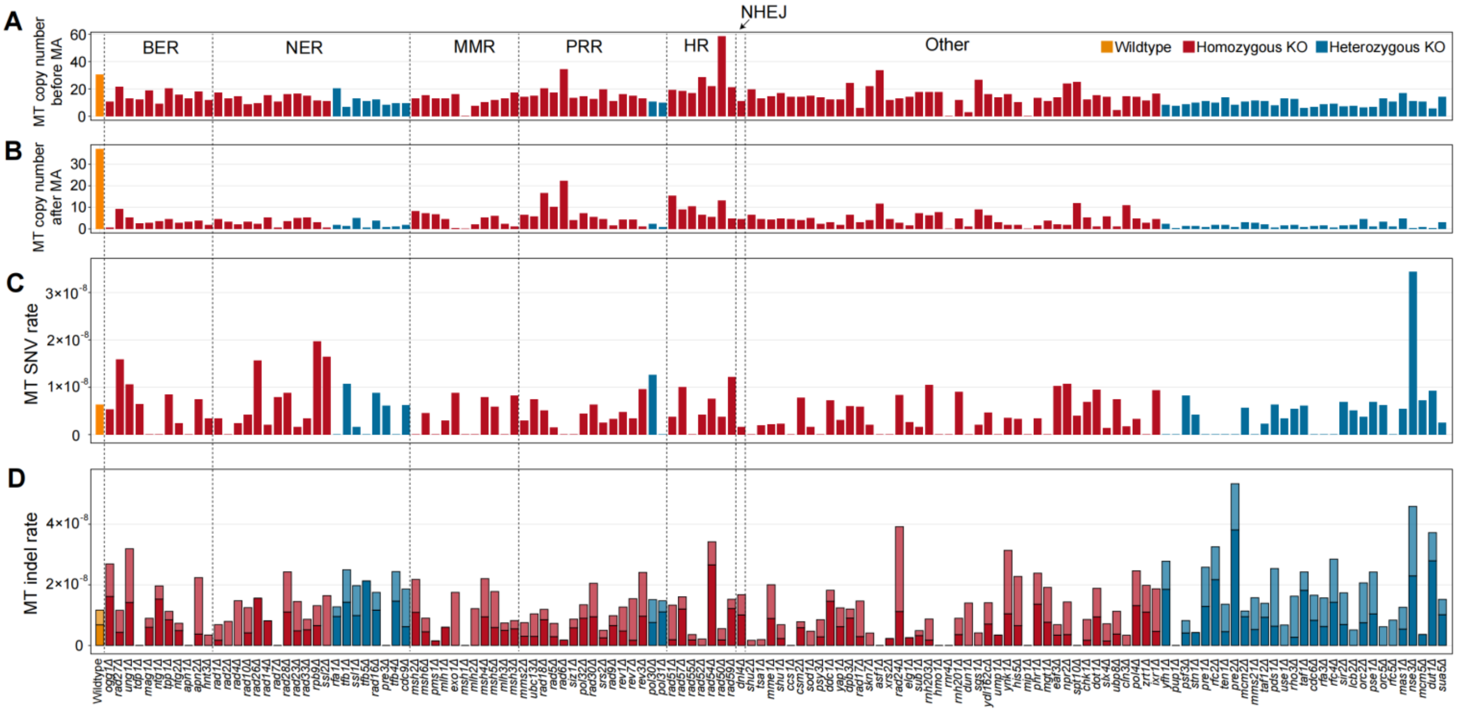

Figure S4. Copy number and mutation rates of mitochondrial genome across the KO strains.

(A) The mitochondrial copy number in the wildtype strain and 136 KO strains prior to the mutation accumulation (MA) experiment.

(B) The average mitochondrial copy number across all replicated MA lines for the wildtype strain and each KO strain.

(C) Single-nucleotide variant (SNV) rates of the mitochondrial genome for the wildtype strain and 136 KO strains.

(D) Insertion and deletion (indel) rates of the mitochondrial genome for the wildtype strain and 136 KO strains. Darker shades indicate insertion rates, while lighter shades represent deletion rates.

Strains are displayed in the same order as in Figure 2, arranged by pathway, KO type, and SNV rate.

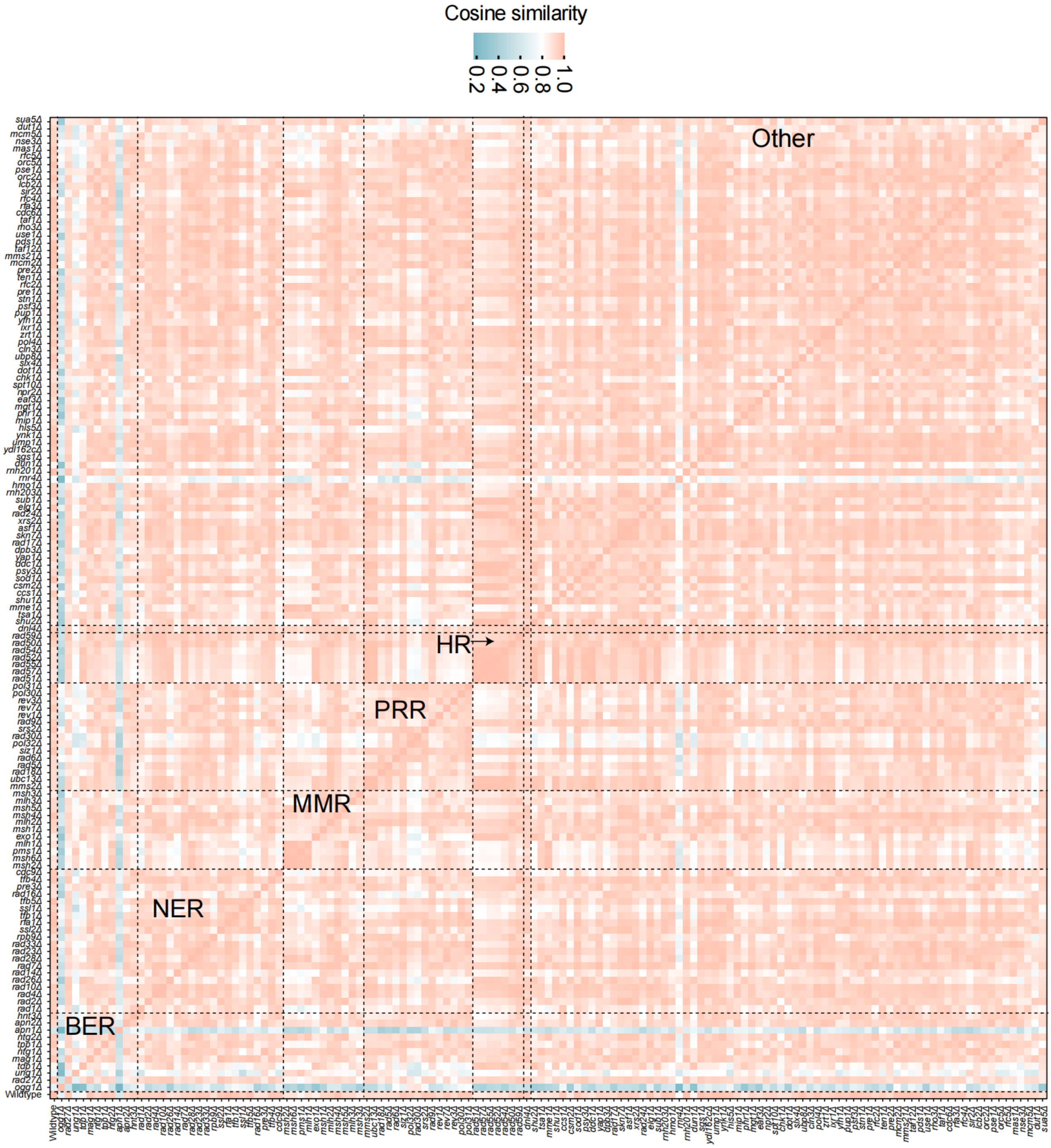

Figure S5. Within- and between-pathway comparisons of correlation in SBS signatures among KO strains. Cosine similarity of the relative frequencies of the six types of SNVs were computed among all KO strains and the WT. Pink indicating higher levels of similarity while blue indicating lower levels of similarity. Pathways are delineated by vertical and horizontal dashed lines. Strains on both the x-axis and y-axis (bottom to top) are arranged in the same order as the x-axis in Figure 2.

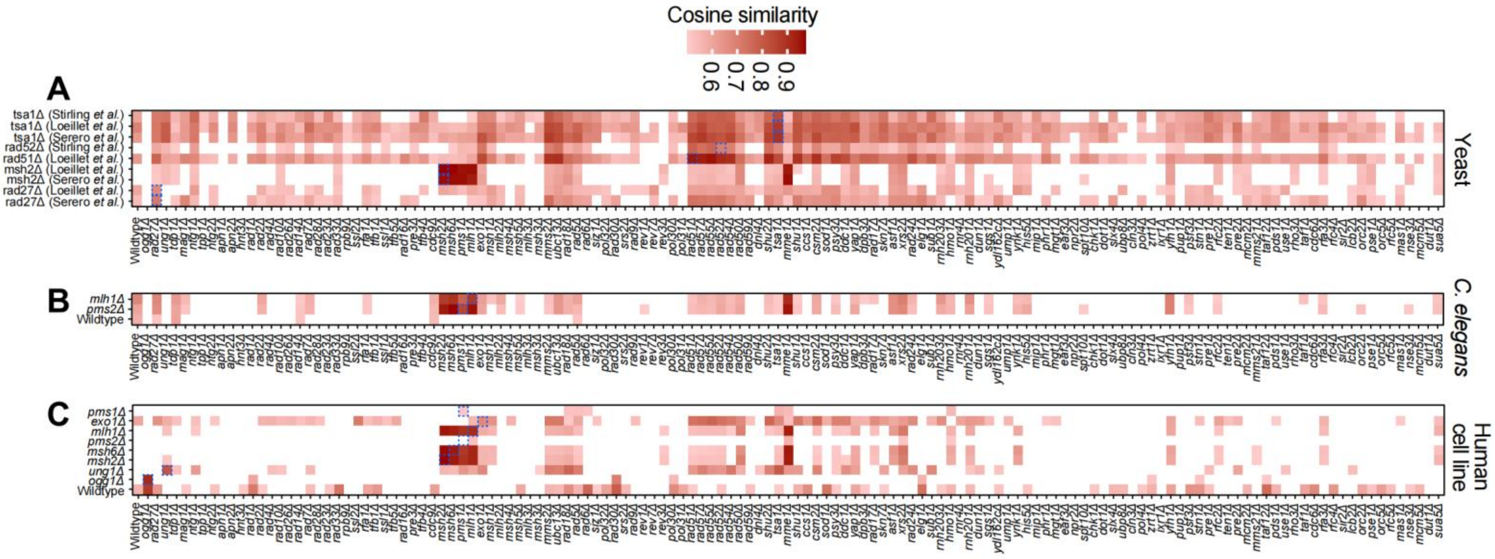

Figure S6. Cosine similarity of the 96 SBS types between this study and previous datasets from budding yeast (A), C. elegans (B), and human cell lines (C). The x-axis indicates knockout strains from this study, and the y-axis shows corresponding data from prior studies. Cosine similarities involving identical strains or orthologous gene deletions are highlighted in blue rectangle.
